# Clonal evolution in relapsed and refractory diffuse large B-cell lymphoma is characterized by high dynamics of subclones

**DOI:** 10.18632/oncotarget.9860

**Published:** 2016-06-06

**Authors:** Thomas Melchardt, Clemens Hufnagl, David M. Weinstock, Nadja Kopp, Daniel Neureiter, Wolfgang Tränkenschuh, Hubert Hackl, Lukas Weiss, Gabriel Rinnerthaler, Tanja N. Hartmann, Richard Greil, Oliver Weigert, Alexander Egle

**Affiliations:** ^1^ Third Medical Department at The Paracelsus Medical University Salzburg, Salzburg, Austria; ^2^ Salzburg Cancer Research Institute (SCRI), Salzburg, Austria; ^3^ Cancer Cluster Salzburg (CCS), Salzburg, Austria; ^4^ Department of Medical Oncology, Dana-Farber Cancer Institute, Boston, MA, USA; ^5^ Institute of Pathology, Paracelsus Medical University Salzburg, Salzburg, Austria; ^6^ Division of Bioinformatics, Biocenter, Medical University of Innsbruck, Innsbruck, Austria; ^7^ University Hospital of The Ludwig-Maximilians-University Munich, Medical Department III, Laboratory for Experimental Leukemia and Lymphoma Research (ELLF), Munich, Germany; ^8^ German Cancer Consortium (DKTK) and German Cancer Research Center (DKFZ), Heidelberg, Germany

**Keywords:** DLBCL, clonal evolution, TP53, tumor heterogeneity, subclonal selection

## Abstract

Little information is available about the role of certain mutations for clonal evolution and the clinical outcome during relapse in diffuse large B-cell lymphoma (DLBCL). Therefore, we analyzed formalin-fixed-paraffin-embedded tumor samples from first diagnosis, relapsed or refractory disease from 28 patients using next-generation sequencing of the exons of 104 coding genes. Non-synonymous mutations were present in 74 of the 104 genes tested. Primary tumor samples showed a median of 8 non-synonymous mutations (range: 0-24) with the used gene set. Lower numbers of non-synonymous mutations in the primary tumor were associated with a better median OS compared with higher numbers (28 versus 15 months, p=0.031). We observed three patterns of clonal evolution during relapse of disease: large global change, subclonal selection and no or minimal change possibly suggesting preprogrammed resistance. We conclude that targeted re-sequencing is a feasible and informative approach to characterize the molecular pattern of relapse and it creates novel insights into the role of dynamics of individual genes.

## INTRODUCTION

Diffuse large B cell lymphoma (DLBCL) is the most common histological subtype of Non-Hodgkin Lymphoma (NHL) and comprises about 20% of newly diagnosed lymphoid neoplasms [1]. Despite chemoimmunotherapy more than a third of patients with DLBCL will experience relapse of disease and the success of second line treatment is significantly worse [2].

Recent research in other hematological diseases has shown that clinical relapse is often caused by the rise of a more aggressive subclone, which was mostly present, but not predominant, at initial diagnosis [3–6]. Despite the fact that multiple mutations have been identified in primary tumor samples of DLBCL [7–12], data about their prognostic role and on the mechanisms of clonal evolution are still scarce. Direct comparison of the primary tumor and relapsed disease is often not feasible in clinical practice due to the lack of a biopsy at relapse and centralized assessment. It is further complicated by technical limitations from formalin fixed tissues. Therefore, we attempt to investigate molecular patterns of relapse using a robust hybrid capture targeted resequencing approach.

## RESULTS

### Patient characteristics

We included 28 DLBCL patients with histologically confirmed relapsed or refractory (r/r) disease in this analysis (patients and tumor characteristics are detailed in Supplementary Table S1 and S2). In summary, advanced disease according to Ann Arbor was present at diagnosis in 68% of the patients and the median age at first diagnosis was 62 years (range 22-84 years).

Cell of origin categories (COO) analysis revealed a germinal center B-cell (GCB) in ten (38%) and a non-GCB phenotype in 16 (62%) out of 26 evaluable patients. *MYC* translocation was present in four of 26 (15%) evaluable cases, two of them had an additional BCL2 rearrangement (Double hit lymphoma) and one patient had additional BCL2 and BCL6 rearrangements (Triple hit lymphoma).

To explore a possible selection bias regarding patients with an available second biopsy we compared these 28 patients with our database of all other DLBCL patients experiencing relapse or refractory disease at our cancer center (n=100). Patients in our sequencing cohort were younger than the other patients (61.1 vs 70.1 years p=0.03) indicating a more aggressive diagnostic approach in younger patients. However, there was no significant difference in progression free survival (PFS) (8 vs. 6 months p=0.10) or IPI and NCCN-IPI risk score between these groups arguing against a major bias factor regarding disease biology.

### Targeted sequencing in primary DLBCL samples

Sequencing was successful in 96.8% of all samples resulting in 25 patients with sequencing of the primary tumor (median coverage: 183X) and 24 patients with available sequence pairs of primary lymphoma and histologically confirmed relapse according to the criteria outlined in the supplementary method section. Overall, non-synonymous mutations were present in 74 of the 104 genes tested (for details see Supplementary Table S3).

**Figure 1:**
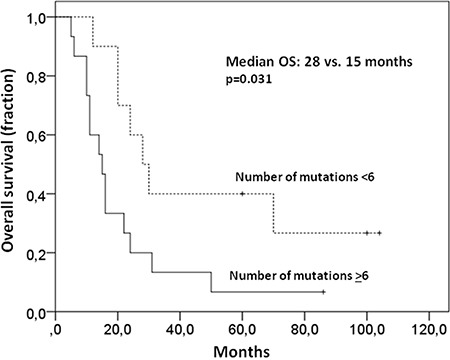
Higher number of mutations in primary samples is associated with a worse OS Less than six non-synonymous mutations in the primary tumor were associated with a better median OS than more mutations (28 versus 15 months p=0.031).

In all but one primary tumor sample at least one non-synonymous mutation (median: 8; range 0-24 mutations) could be identified using a targeted sequencing approach (see Supplementary Figure S1). As determined by Receiver Operating Characteristic calculation and Youden Index analysis less than six non-synonymous mutations in the primary tumor were associated with a better median overall survival (OS) compared with more mutations (28 versus 15 months p=0.031; Figure 1) in our cohort. PFS, however, was not affected (10 versus 7 months p=0.14) by the number of mutations, suggesting an influence of mutation burden on the ability to salvage relapsed disease. Known adverse prognostic factors such as high IPI, non-GCB phenotype or MYC status were not associated with the number of non-synonymous mutations in the primary tumor.

Estimation of clonal heterogeneity in DLBCL using targeted resequencing has not been reported so far. We assessed the presence of subclones from an analysis of allelic frequencies to estimate clonal heterogeneity within the primary tumor using all the somatic mutations in the primary samples. Due to lack of standardized assessment tools we arbitrarily defined subclonal disease, indicating clonal heterogeneity within the primary sample, as existence of a clone with an allelic fraction (AF) lower than the 25^th^ percentile (20.4%) of the AF of all mutations in the primary samples. Such a subclonal population within the primary tumor indicating clonal tumor heterogeneity was found in 21 of 25 (84%) samples. However, this was neither associated with clinical outcome (PFS: 8 vs 15 months p=0.62; OS: 16 vs 24 months p=0.93) nor with COO or *MYC*-status. Nevertheless, all sample pairs allowed to confirm the clonal identity of primary and relapsed disease by observation of common mutations arguing against a second independent clone.

### Mode of clonal evolution

The nature of clonal evolution associated with relapse or refractoriness of DLBCL is unknown so far. Immunohistochemical parameters and translocations have been reported to be stable over the course of disease in DLBCL [13], but analyses on mutational level are pending.

**Figure 2:**
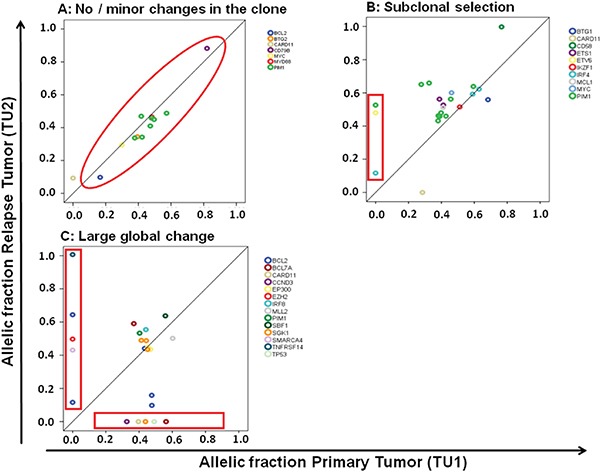
Different molecular patterns of relapse in DLBCL **C.** A pattern of a large global change at the timepoint of relapse was observed in 15 of 24 (62.5%) patients and a more stable pattern in 7 of 24 patients (29.1%). The latter one can be further divided in a pattern of no or minor changes of the malignant clone **A.** and a pattern of subclonal selection **B.**

We observed relevant dynamics on mutational level in almost all patients using targeted next generation sequencing. We categorized relapses by patterns observed. We defined as large global change at the time point of relapse the complete loss of a mutation labeling the dominating tumor population at first diagnosis (allelic fraction higher than 45%). Such large global changes were observed in 15 of 24 (62.5%) evaluable patients. A more stable pattern without loss of the predominant clone was present in 7 of 24 (29.1%) patients (Figure 2). Two cases were classified as “indeterminate”. These observed evolutions with a large global changing or stable pattern were not associated with the clinical outcome (median PFS: 8 vs 15 months p=0.42; median OS: 16 vs 24 months p=0.46) nor clinical risk scores or COO phenotype. The existence of subclonal disease in the primary sample was associated with the evolutionary pattern of large global change at relapse (p=0·04). Interestingly, all four cases with a *MYC* translocation showed a pattern of a large global change, but this association did not reach statistical significance (p=0.11).

When further analyzing the 7 patients showing a stable pattern without loss of the predominant clone, we observed in 5 patients a gain of new mutations with an AF of at least 40% at relapse suggesting selection of a new clone while in two patients no gain of new mutations was observed. This may either suggest the presence of preprogrammed resistance of the malignant clone or relevant evolution outside the targeted gene set (Figure 2). Categorizing these three types of clonal evolution (large global change, subclone selection, and minimal change due to preprogrammed resistance) there was no significant association with clinical endpoints and risk scores, or molecular markers (COO, *MYC*).

### Dynamic of subclones shown by mutated PIM1 and BCL2 during disease progression

**Figure 3:**
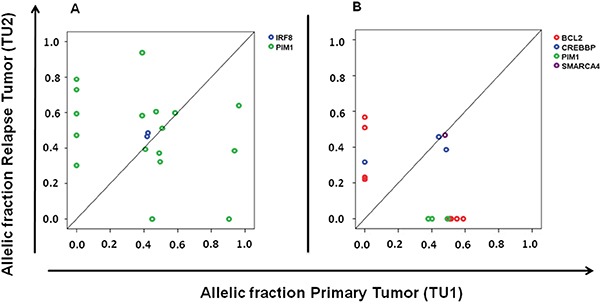
Dynamics of subclones marked by PIM1 and BCL2 mutations during disease progression **A.** Comparison of primary and subsequent tumor sample show the gain of five and the loss of two PIM1 mutations; **B.** Gain of four *BCL2* mutations and the loss of three *PIM1* and four *BCL2* mutations. It is notable that the major tumor clone characterized by other mutations (*IRF 8* Figure 3A; *CREBBP* and *SMARCA4* Figure 3B) did not change over time.

In addition to the high rate of *PIM1* (nine of 25 samples) and *BCL2* mutated cases (nine of 25 samples; Supplementary Table S3) we observed multiple mutations per case of these genes in the majority of cases, as would be predicted from their known role as target of somatic hypermutation [14]. We, however, noted a high rate of loss and gain of the allelic fractions of individual mutations within these two genes during disease indicating high clonal dynamics within these tumors, while the allelic fractions of the major tumor cell fraction marked by disease defining mutations did not change (two representative samples in Figure 3). These findings indicate a dynamic process of fluctuating of smaller clones during evolution and therapy in DLBCL that was marked by *PIM1* and *BCL2* mutations, suggesting that hypermutated genes may be useful to track clonal evolution.

While *BCL2* hypermutation (defined as more than one non-synonymous mutation) was associated with a GCB phenotype (five of ten GCB vs one of 14 non-GCB cases, p=0.017), *PIM1* hypermutation was more prevalent albeit not statistically significant in non-GCB cases (five of 14 non-GCB vs one of 10 GCB cases; p=0.15). This lines up with previous reports of these genes [11, 14, 15].

### Mutation frequency in primary and relapsed / refractory tumors

As an exploratory analysis we compared the frequency of mutated genes in our cohort with a gene list compiled from 6 major reports containing patients with extensive sequencing (up to 458 patients per gene). Due to the lack of complete clinical data and follow-up in these reports [7–12] the clinical impact of the described mutations is still unknown. Commonly mutated genes such as *CARD11, CD58, CD79B, CREBBP, EZH2, MYD88,* or *B2M* showed no difference in mutation frequency in our patients selected for relapse, when compared to the reported presumably less selected 6 cohorts. Nevertheless, despite low numbers in our cohort some mutations previously reported to be rare or absent in DLBCL were significantly more often observed in our primary samples (*NOTCH1, MYC, RB1, FAT2, ATM, SMARCA4, MCL1*) and relapsed samples (*TP53, MCL1, ATM, FAT2, MYC, RB1, SMARCA4*) (see Supplementary Tables S3 and S6) when compared to the literature of primary samples [7–12].

### Dynamics of individual mutations over time

**Figure 4:**
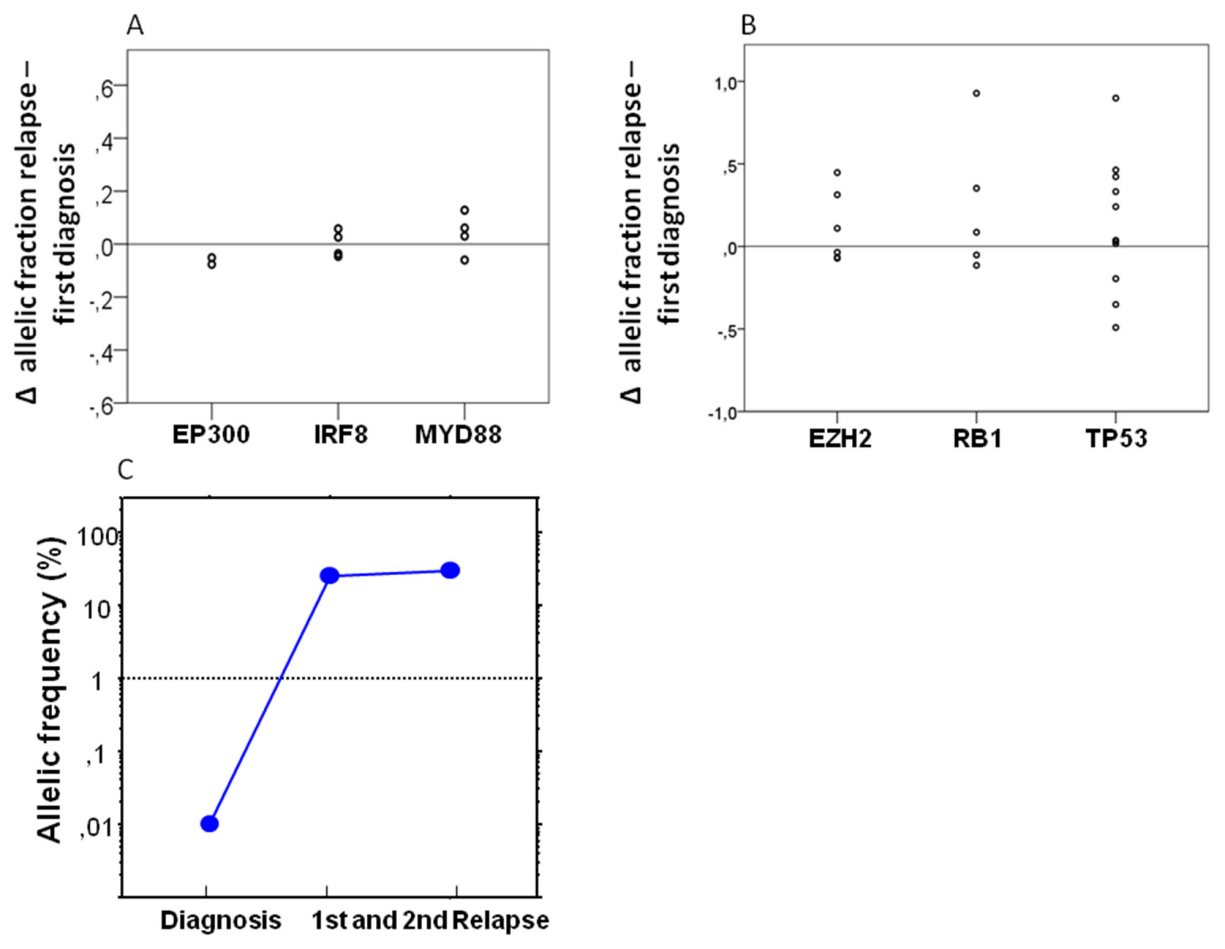
Dynamics of individual genes over time **A.** Analyzing the dynamics of individual genes we observed a completly stable pattern of the genes *EP300* (two mutations in two patients), *IRF8* (four mutations in three patients) and *MYD88* (four mutations in four patients) analyzing the difference of the allelic fractions of the primary and relapse tumor. **B.** A relevant gain of the allelic fraction was observed in the *TP53* gene in three patients (five mutations), in the *RB1* gene in one patient (one mutation) and in the *EZH2* gene in two patients (two mutations). **C.** To prove the existence of small *TP53* mutated subclones in the primary samples before chemotherapy we also performed ultra-deep sequencing. We were able to detect a small subclone with an AF 0_·_1% with a median coverage of 51,830X.

We were interested to see whether our limited set of genes would allow exploring the evolutionary patterns of individual genes. We found genes that displayed a stable behavior in all observed cases, compatible with a possible role as truncal mutations e.g.: *EP300* (two mutations in two patients), *IRF8* (four mutations in three patients), and *MYD88* (four mutations in four patients, see also Figure 4).

Nevertheless, in the majority of cases and genes relevant dynamics were observed. The most obvious pattern was acquisition of mutations in relapse. As could be expected from its association with poor prognosis we found gain of *TP53* mutations in three patients, but we also observed clearly novel mutations appearing in relapse e.g.: mutation of the *RB1* gene in one patient (one mutation) and in the *EZH2* gene in two patients (two mutations, see Figure 4).

*TP53* mutations were shown to be present at low frequency in CLL samples before relapse with a dominating p53 deficient clone [16]. To investigate the existence of small *TP53* mutated subclones in the primary DLBCL samples we performed ultra-deep sequencing of these mutations in two patients. We were able to detect in both patients a small subclones with an AF of 0.45% and 0.1% with a median coverage of 51,830X (see also Figure 4C), which were below of detection levels of our conventional NGS approach. These findings suggest a selection rather than new generation of *TP53* mutated tumor cells by R-CHOP.

We, however, also found mutations in known DLBCL-associated genes to be lost in relapse. For example, we found *CARD11* in three of the 25 (12%) primary tumor samples, roughly matching the expected rate. Surprisingly, we could not detect these mutations in the matched samples of r/r disease in two of these three patients by conventional NGS suggesting that the *CARD11* mutated clone was not truncal. Using this approach we also observed a loss of initially detected *CREBBP* mutations in the samples of r/r disease in three of five patients. We also performed targeted ultra-deep sequencing of these *CARD11* and *CREBBP* mutations and could detect residual small subclones in both relapsed *CARD11* samples (AF: 0.06% and 0.02%; median coverage: 430,000X) and two *CREBBP* samples (AF: 0.2% and 0.1%; median coverage: 500,000X).

## DISCUSSION

In our project we used targeted next generation sequencing of 104 genes known to be frequently mutated in NHL to analyze a homogenously treated cohort of 28 DLBCL patients, who experienced r/r disease.

Due to the nature of targeted resequencing our approach is blind beyond the selected genes and it was unclear whether a limited gene set of around 100 genes could be used to uncover patterns of evolution. Whole exome or genome sequencing would be desirable, but these approaches are optimized for fresh-frozen biopsies, which are not available in the majority of DLBCL patients in clinical routine and exome sequencing from formalin-fixed, paraffin-embedded tissues remains a challenge. We show that an approach using targeted resequencing is feasible to detect a meaningful number of mutations and small subclones within one tumor, and to track clonal evolution in FFPE samples of DLBCL.

First, we were interested if measures of clonal complexity of the primary lymphoma were associated with clinical outcome. Overall tumor heterogeneity defined by the presence of smaller subclones was not associated with survival or other clinical characteristics. However, using the number of non-synonymous mutations derived from our gene set as surrogate for genetic complexity, we found that patients with less than six non-synonymous mutations had a better OS than the other patients, (see Figure 1), possibly indicating a more favourable biology in DLBCL clones with a lower number of mutations.

**Figure 5:**
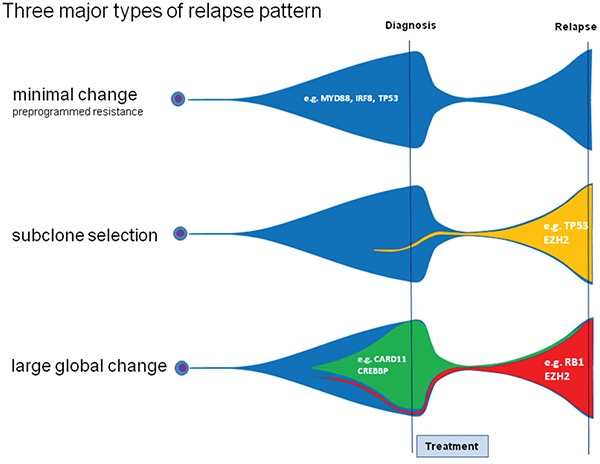
Three different relapse patterns in DLBCL Analyzing patterns of primary and relapsed diseases we observed distinct patterns of relapse. The pattern of large global change is characterized by the complete loss of the dominating clone. The other patterns are characterized by no/minor changes in the malignant clone due to preprogrammed resistance and can be accompanied by the rise of a new dominating clone.

We successfully identified a mode of clonal evolution in 92% of evaluable cases. The three major patterns of clonal evolution we describe (see Figure 2 and 5) are paralleled in a recent study using VDJ rearrangement to track evolution in 14 matched samples of primary and relapsed DLBCL. These authors proposed an early-divergent and a late-divergent mode of clonal evolution [17], similar to our classification. Importantly, our data make it seem difficult to envision appropriate tools to predict the mechanisms of clonal evolution from the baseline sample in the near future. Interestingly, our data also provide the first description of this phenomenon in a small set of DLBCL with double- or triple-hit phenotype and suggest that also in these cases relapses stem from small subclones that will be difficult to detect in the primary sample.

We then analyzed the role of known driver mutations in DLBCL. *TP53* mutations are known to be involved in refractoriness to chemotherapy in DLCBL, but their dynamics in clonal evolution have not been reported. We found a clonal selection from very small pre-existing subclones in our sample set suggesting a selection of pre-existing *TP53* mutations rather than novel mutations during administered chemotherapy. This is in line with reports in CLL [16].

Targeted sequencing approaches have a limited capability to identify novel mutations, but the loss of previously identified mutations provides reliable insights into clonal evolution. Interestingly, we found loss of mutations clearly suggested to be important for the pathogenesis of lymphoma. We observed the loss of several *CARD11* and *CREBBP* mutations during progression of DLBCL to subclonal level in r/r samples. In the case of two COSMIC annotated *CARD11* mutations, lost to subclonal level, this observation fits well with the previous published report about the varying biological influence of different *CARD11* mutations [18], suggesting that functional information needs to be considered in all cases. As an anecdote, we also observed the loss of a *TP53* mutation in relapse (data not shown). This patient displayed new mutations of *SMARCA4* and *EZH2* in the relapse samples suggesting a potential role for relapse independent of *TP53.* Truncal mutations allowed assuring the identity of the founder clone in all cases. These findings of loss and gain of mutations during clonal evolution challenge the concept of non-invasive disease monitoring by quantification of circulating tumor-DNA based on the mutational spectrum of the primary tumor.

Using a comparison of the mutational spectrum established in our cohort selected for relapse with a gene set established from the published literature on less selected baseline samples, we were able to point to a few novel candidates for determining resistance in DLCBL. Aside from the expected *TP53* mutations we also established a list of genes that could be targets for further validation as determinants of resistance. Among them *RB1* or *ATM* have a relatively clear rational and were reported be associated with poor prognosis in other types of cancer [19–23], but genes such as *FAT2* or *SMARCA4* may represent novel resistance genes. Interestingly, *FAT1* has been reported in resistance in CLL and *SMARCA4* was associated with Burkitt lymphoma [24, 25]. Larger studies as well as functional studies will be necessary to validate our proposed candidates.

In conclusion, we describe the major patterns of clonal evolution in DLBCL using targeted resequencing. This approach was not only feasible but also informative in creating insights into the role of dynamics of individual genes and suggestive of resistance associated genes for further validation.

## PATIENTS AND METHODS

### Patients

We identified 28 patients with histologically confirmed r/r DLBCL and sufficient amount of formalin-fixed, paraffin-embedded tumor tissue from a retrospective analysis of 128 relapsed DLBCL cases observed in 317 patients consecutively diagnosed at the Third Medical Department of the Paracelsus Medical University Salzburg between 2003 and 2013. Tumor cell content was measured by immunohistochemial as well as hematoxylin and eosin staining. COO categories, GCB and non-GCB, were classified according to the Hans algorithm [26]. *MYC* interphase FISH was performed on standard tissue sections using *MYC* FISH, Split Signal Code Y5410 (Dako Denmark A/S, Glostrup) and ZytoLight SPEC BCL2 / 6 Dual Color Break Apart Prob (Zytovision Germany).

Clinical characteristics, OS and PFS after first line therapy were retrospectively analyzed by chart based review. For patients, who did not attend to follow-up visits, clinical follow-up data were obtained by telephone interviews with the patients' general practitioner.

This analysis was approved by the Ethics Committee of the provincial government of Salzburg, Austria (415-E/1540/4-2013) and written informed consent was obtained.

### Targeted next-generation sequencing

Primary formalin-fixed, paraffin-embedded tumor samples, samples of refractory or relapsed disease, and matched germ line were used for next generation sequencing. Targeted exon capture, sequencing of all coding exons of 104 selected genes known to be frequently mutated in lymphoma, were performed as previously described [27, 28] (for gene list see Supplementary Table S4). A matched non-tumor sample, mostly from morphologically unaffected bone marrow, was evaluable in 93% of patients and was used to exclude germ-line polymorphisms. Identities of tumor and matched individual germ-line DNA samples were additionally confirmed by fingerprint genotyping [29]. The measured allelic frequencies were normalized to the assessed tumor cell content. For validation we performed target re-sequencing of the previously detected mutation in 5 patients (see Supplementary Figure S2 for details) using a Ion Torrent plattform. We achieved a validation rate of 97.6% of 82 tested mutations and an excellent reproducibility of the allelic fractions tested correlation (Pearson's correlation coefficient: 0.91; p<0.01).

### Ultra-deep sequencing of selected mutations

Mutations of the *TP53, CARD11,* and *CREBBP* genes were selected for further sequencing with higher coverage to detect small subclones. We designed sequences that flank the targeted regions of interest (Supplementary Table S5) and the amplified sequences were isolated after gel electrophoresis. Targeted resequencing of the sample of interest, the matched germ line sample as negative and the corresponding tumor sample with already established mutation as positive control was performed on a MiSeq (Illumina) system using the reagent kit v2 2*250.

### Statistical analysis

Statistical analyses were performed using IBM^®^ SPSS^®^ statistics software, version 21 and R-statistical software environment (including packages *survival*, *CPE*). Mann-Whitney-U-test, Pearson's chi-squared test, and Fisher's exact test were used for univariate analyses, where appropriate. Survival was estimated using Kaplan-Meier curve analysis, with statistical comparison using the log-rank test. A two-tailed significance-level of 0.05 was considered statistically significant. The cut-off values in our cohort were determined by Receiver Operating Characteristic calculation and Youden Index analysis for OS.

## SUPPLEMENTARY DATA, SUPPLEMENTARY MATERIALS AND METHODS



## References

[1] Morton LM, Wang SS, Devesa SS, Hartge P, Weisenburger DD, Linet MS (2006). Lymphoma incidence patterns by WHO subtype in the United States, 1992-2001. Blood.

[2] Gisselbrecht C, Glass B, Mounier N, Singh GD, Linch DC, Trneny M, Bosly A, Ketterer N, Shpilberg O, Hagberg H, Ma D, Briere J, Moskowitz CH (2010). Salvage regimens with autologous transplantation for relapsed large B-cell lymphoma in the rituximab era. J Clin Oncol.

[3] Ding L, Ley TJ, Larson DE, Miller CA, Koboldt DC, Welch JS, Ritchey JK, Young MA, Lamprecht T, McLellan MD, McMichael JF, Wallis JW, Lu C (2012). Clonal evolution in relapsed acute myeloid leukaemia revealed by whole-genome sequencing. Nature.

[4] Wang L, Lawrence MS, Wan Y, Stojanov P, Sougnez C, Stevenson K, Werner L, Sivachenko A, DeLuca DS, Zhang L, Zhang W, Vartanov AR, Fernandes SM (2011). SF3B1 and other novel cancer genes in chronic lymphocytic leukemia. N Engl J Med.

[5] Rossi D, Rasi S, Spina V, Bruscaggin A, Monti S, Ciardullo C, Deambrogi C, Khiabanian H, Serra R, Bertoni F, Forconi F, Laurenti L, Marasca R (2013). Integrated mutational and cytogenetic analysis identifies new prognostic subgroups in chronic lymphocytic leukemia. Blood.

[6] Landau DA, Carter SL, Stojanov P, Mckenna A, Stevenson K, Lawrence MS, Sougnez C, Stewart C, Sivachenko A, Wang L, Wan Y, Zhang W, Shukla SA (2013). Evolution and impact of subclonal mutations in chronic lymphocytic leukemia. Cell.

[7] Lawrence MS, Stojanov P, Mermel CH, Robinson JT, Garraway LA, Golub TR, Meyerson M, Gabriel SB, Lander ES, Getz G (2014). Discovery and saturation analysis of cancer genes across 21 tumour types. Nature.

[8] Lohr JG, Stojanov P, Lawrence MS, Auclair D, Chapuy B, Sougnez C, Cruz-Gordillo P, Knoechel B, Asmann YW, Slager SL, Novak AJ, Dogan A, Ansell SM (2012). Discovery and prioritization of somatic mutations in diffuse large B-cell lymphoma (DLBCL) by whole-exome sequencing. Proc Natl Acad Sci U S A.

[9] Morin RD, Mendez-Lago M, Mungall AJ, Goya R, Mungall KL, Corbett RD, Johnson NA, Severson TM, Chiu R, Field M, Jackman S, Krzywinski M, Scott DW (2011). Frequent mutation of histone-modifying genes in non-Hodgkin lymphoma. Nature.

[10] Morin RD, Mungall K, Pleasance E, Mungall AJ, Goya R, Huff RD, Scott DW, Ding J, Roth A, Chiu R, Corbett RD, Chan FC, Mendez-Lago M (2013). Mutational and structural analysis of diffuse large B-cell lymphoma using whole-genome sequencing. Blood.

[11] Pasqualucci L, Trifonov V, Fabbri G, Ma J, Rossi D, Chiarenza A, Wells VA, Grunn A, Messina M, Elliot O, Chan J, Bhagat G, Chadburn A (2011). Analysis of the coding genome of diffuse large B-cell lymphoma. Nat Genet.

[12] Zhang J, Grubor V, Love CL, Banerjee A, Richards KL, Mieczkowski PA, Dunphy C, Choi W, Au WY, Srivastava G, Lugar PL, Rizzieri DA, Lagoo AS (2013). Genetic heterogeneity of diffuse large B-cell lymphoma. Proc Natl Acad Sci U S A.

[13] Thieblemont C, Briere J, Mounier N, Voelker HU, Cuccuini W, Hirchaud E, Rosenwald A, Jack A, Sundstrom C, Cogliatti S, Trougouboff P, Boudova L, Ysebaert L (2011). The Germinal Center/Activated B-Cell Subclassification Has a Prognostic Impact for Response to Salvage Therapy in Relapsed/Refractory Diffuse Large B-Cell Lymphoma: A Bio-CORAL Study. J Clin Oncol.

[14] Pasqualucci L, Neumeister P, Goossens T, Nanjangud G, Chaganti RS, Kuppers R, la-Favera R (2001). Hypermutation of multiple proto-oncogenes in B-cell diffuse large-cell lymphomas. Nature.

[15] Pasqualucci L, la-Favera R (2014). SnapShot: diffuse large B cell lymphoma. Cancer Cell.

[16] Rossi D, Khiabanian H, Spina V, Ciardullo C, Bruscaggin A, Fama R, Rasi S, Monti S, Deambrogi C, De PL, Wang J, Gattei V, Guarini A (2014). Clinical impact of small TP53 mutated subclones in chronic lymphocytic leukemia. Blood.

[17] Jiang Y, Redmond D, Nie K, Eng KW, Clozel T, Martin P, Tan LH, Melnick AM, Tam W, Elemento O (2014). Deep sequencing reveals clonal evolution patterns and mutation events associated with relapse in B-cell lymphomas. Genome Biol.

[18] Lenz G, Davis RE, Ngo VN, Lam L, George TC, Wright GW, Dave SS, Zhao H, Xu W, Rosenwald A, Ott G, Muller-Hermelink HK, Gascoyne RD (2008). Oncogenic CARD11 mutations in human diffuse large B cell lymphoma. Science.

[19] Beggs AD, Domingo E, McGregor M, Presz M, Johnstone E, Midgley R, Kerr D, Oukrif D, Novelli M, Abulafi M, Hodgson SV, Fadhil W, Ilyas M (2012). Loss of expression of the double strand break repair protein ATM is associated with worse prognosis in colorectal cancer and loss of Ku70 expression is associated with CIN. Oncotarget.

[20] Santarpia L, Iwamoto T, Di LA, Hayashi N, Bottai G, Stampfer M, Andre F, Turner NC, Symmans WF, Hortobagyi GN, Pusztai L, Bianchini G (2013). DNA repair gene patterns as prognostic and predictive factors in molecular breast cancer subtypes. Oncologist.

[21] Kim H, Saka B, Knight S, Borges M, Childs E, Klein A, Wolfgang C, Herman J, Adsay VN, Hruban RH, Goggins M (2014). Having pancreatic cancer with tumoral loss of ATM and normal TP53 protein expression is associated with a poorer prognosis. Clin Cancer Res.

[22] Zhao W, Huang CC, Otterson GA, Leon ME, Tang Y, Shilo K, Villalona MA (2012). Altered p16(INK4) and RB1 Expressions Are Associated with Poor Prognosis in Patients with Nonsmall Cell Lung Cancer. J Oncol.

[23] Chano T, Ikebuchi K, Tomita Y, Jin Y, Inaji H, Ishitobi M, Teramoto K, Ochi Y, Tameno H, Nishimura I, Minami K, Inoue H, Isono T (2010). RB1CC1 together with RB1 and p53 predicts long-term survival in Japanese breast cancer patients. PLoS One.

[24] Messina M, Del G, Khiabanian H, Rossi D, Chiaretti S, Rasi S, Spina V, Holmes AB, Marinelli M, Fabbri G, Piciocchi A, Mauro FR, Guarini A (2014). Genetic lesions associated with chronic lymphocytic leukemia chemo-refractoriness. Blood.

[25] Love C, Sun Z, Jima D, Li G, Zhang J, Miles R, Richards KL, Dunphy CH, Choi WW, Srivastava G, Lugar PL, Rizzieri DA, Lagoo AS (2012). The genetic landscape of mutations in Burkitt lymphoma. Nat Genet.

[26] Hans CP, Weisenburger DD, Greiner TC, Gascoyne RD, Delabie J, Ott G, Muller-Hermelink HK, Campo E, Braziel RM, Jaffe ES, Pan Z, Farinha P, Smith LM (2004). Confirmation of the molecular classification of diffuse large B-cell lymphoma by immunohistochemistry using a tissue microarray. Blood.

[27] Odejide O, Weigert O, Lane AA, Toscano D, Lunning MA, Kopp N, Kim S, van BD, Bolla S, Schatz JH, Teruya-Feldstein J, Hochberg E, Louissaint A (2014). A targeted mutational landscape of angioimmunoblastic T-cell lymphoma. Blood.

[28] Pastore A, Jurinovic V, Kridel R, Hoster E, Staiger AM, Szczepanowski M, Pott C, Kopp N, Murakami M, Horn H, Leich E, Moccia AA, Mottok A (2015). Integration of gene mutations in risk prognostication for patients receiving first-line immunochemotherapy for follicular lymphoma: a retrospective analysis of a prospective clinical trial and validation in a population-based registry. Lancet Oncol.

[29] Demichelis F, Greulich H, Macoska JA, Beroukhim R, Sellers WR, Garraway L, Rubin MA (2008). SNP panel identification assay (SPIA): a genetic-based assay for the identification of cell lines. Nucleic Acids Res.

