# Supplementary material for: Clonal evolution in relapsed and refractory diffuse large B-cell lymphoma is characterized by high dynamics of subclones
Source: Oncotarget. 2016 Jun 6;7(32):51494–502. doi: 10.18632/oncotarget.9860 (PMC5239491; doi:10.18632/oncotarget.9860)
Supplement: Supplementary file 1 [file oncotarget-07-51494-s001.pdf]

Clonal evolution in relapsed and refractory diffuse large B-cell lymphoma is characterized by high dynamics of subclones

SUPPLEMENTARY DATA

SUPPLEMENTARY MATERIALS AND METHODS

Sequencing library preparation

As part of a cooperative lymphoma sequencing approach DNA was isolated from 5-10 x 10 µm sections from FFPE biopsies using QIAamp DNA FFPE Tissue Kit (Qiagen). Quant-iT™ PicoGreen® dsDNA (Invitrogen) was used for quantification. 200 ng DNA was fragmented (Covaris sonication) to 250 bp and further purified using Agentcourt AMPureXP beads (Beckman Coulter). Size-selected DNA was then ligated to specific dual index adaptors during library preparation (SPRIworksHT, Beckman-Coulter). Each library was quantified using qPCR and analysed for quality after fragmentation and library preparation based on library yield and size on an Agilent Bioanalyzer. Samples with yields below 200 ng were excluded from sequencing.

Libraries were mixed in pools of 20-48 samples each, in equal mass to a total of 500 ng per pool and enriched for genes using the PLG_FL1.0 targeted panel as previously described (Pastore et al. 2015) and the Agilent SureSelect hybrid capture kit (see supplementary table 4 for details). Each capture pool was sequenced on one lane of the HiSeq 2500 in Rapid Run Mode.

Tumor cell content

Tumor cell content measured by immunohistochemial as well as hematoxylin and eosin staining was assessed by two experienced hematopathologists (D.N. and W.T.) with a median content of 80% (range 30-100%).

Pre-analysis processing

Pooled sample reads were de-convoluted (de-multiplexed) and sorted using the Picard tools (see http://picard.sourceforge.net/command-line-overview.shtml for details). Reads were aligned to thereference sequence b37 edition from the Human Genome Reference Consortium using bwa (http://biobwa.sourceforge.net/bwa.shtml) using the following parameters "-q 5 -l 32 -k 2 -o 1" and duplicate readswere identified and removed using the Picard tools.

The alignments were refined using the GATK tool for localized realignment around indel sites (http://gatkforums.broadinstitute.org/discussion/38/local-realignment-around-indels). Recalibration of the quality scores was also performed using GATK tools (http://gatkforums.broadinstitute.org/discussion/44/base-quality-score-recalibration-bqsr).

Quality control for sequencing

The minimum quality criterion was 80% of target bases have > 30x sequencing coverage. Cases with 60-79% of target bases with > 30x sequencing coverage were also included if target bases not covered was <0.6%. Cases with target bases covered 30x < 60% or cases with target bases covered 30x between 60-80% and target bases not covered > 0.6% were excluded.

Variant analysis

Mutation analysis for single nucleotide variants (SNV) was performed using MuTect v1.1.42 and annotatedby Oncotator (http://www.broadinstitute.org/oncotator/). Insertions and deletions (InDels) were calledusing Indel Locator (http://www.broadinstitute.org/cancer/cga/indelocator).

SUPPLEMENTARY RESULTS

Sequencing results

As already stated sequencing was successful in 96.8% of all samples. Non-synonymous mutations were present in 74 of the 104 genes tested. We detected 697 exonic mutations in the tumor samples overall, consisting of 21.1% silent, 66.7% missense or non-sense, 7.9% frame shift and 4.3% splice site mutations. Detailed information about the locus of mutations known to be important and frequent in DLBCL is listed in Supplemental Table S6.

Validation of the sequencing results

For confirmation we performed validation of 5 (ID 8, 13, 24, 48, 49) patients from on a different platform (Ion Torrent PGM sequencer) using a newly designed bait

set and aiming for higher read numbers. These 5 patients were selected based on different patterns of evolution (2 patterns of no change and 3 patterns of large global change), high number of mutations and availability of remaining DNA.

When using this new customized gene panel based on the previous results we obtained an about 4-fold higher sequencing depth (median coverage: 591X). We achieved a validation rate of 97.6% of 82 tested mutations using the Ion Torrent platform. We also compared the allelic fractions of the 80 successfully tested mutations in the tumor samples between both NGS approaches. This analysis showed an excellent reproducibility of our results with a very high correlation (Pearson's correlation coefficient: 0.91; $p<0.01$) and of the observed patterns of evolution (see also Supplement Figure 2).

Clonal relationship of the tumor samples

Based on shared mutations we detected a common mutation in all 24 patients feasible for evolution analyses suggesting. Furthermore, we selected 4 patients out of these for further VDJ rearrangement analyses (ID 11, 39, 40 and 48) based on a long time to next biopsy (median: 36 months, range 22-80 months). We detected identical rearrangements using sequencing in both tumor samples of 3 patients (ID 11, 40 and 48) and two rearrangements with VH 6 in both tumor samples of patient ID39. The occurrence of two rearrangements in the latter one obviated a valid sequencing result.

As additional evidence for clonal relationship we observed shared mutations of all three samples with a median number of 4 single nucleotide variants (range 2-25) in all 9 patients (Pat ID 2, 8, 12, 13, 17, 27, 30, 36, 37) with two available biopsies of relapsed or refractory disease.

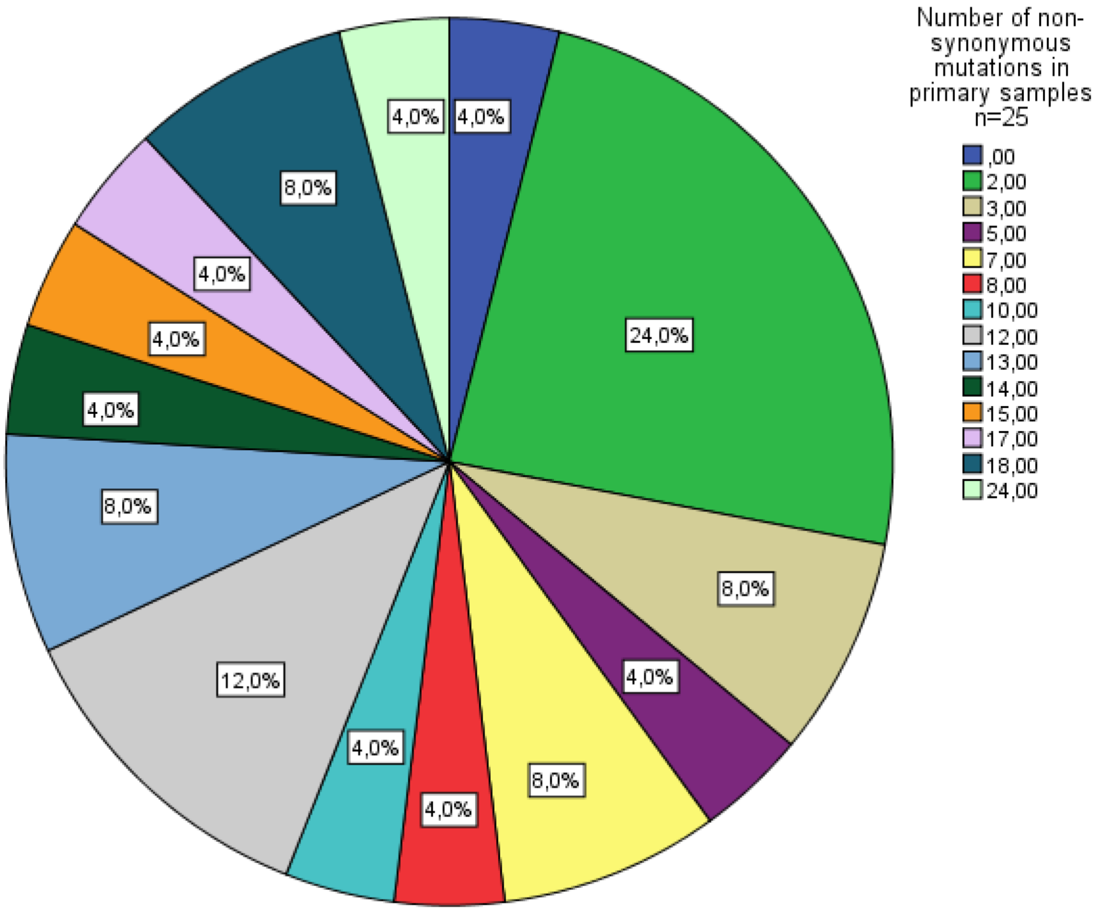

Supplementary Figure S1: Number of non-synonymous mutations in primary samples.

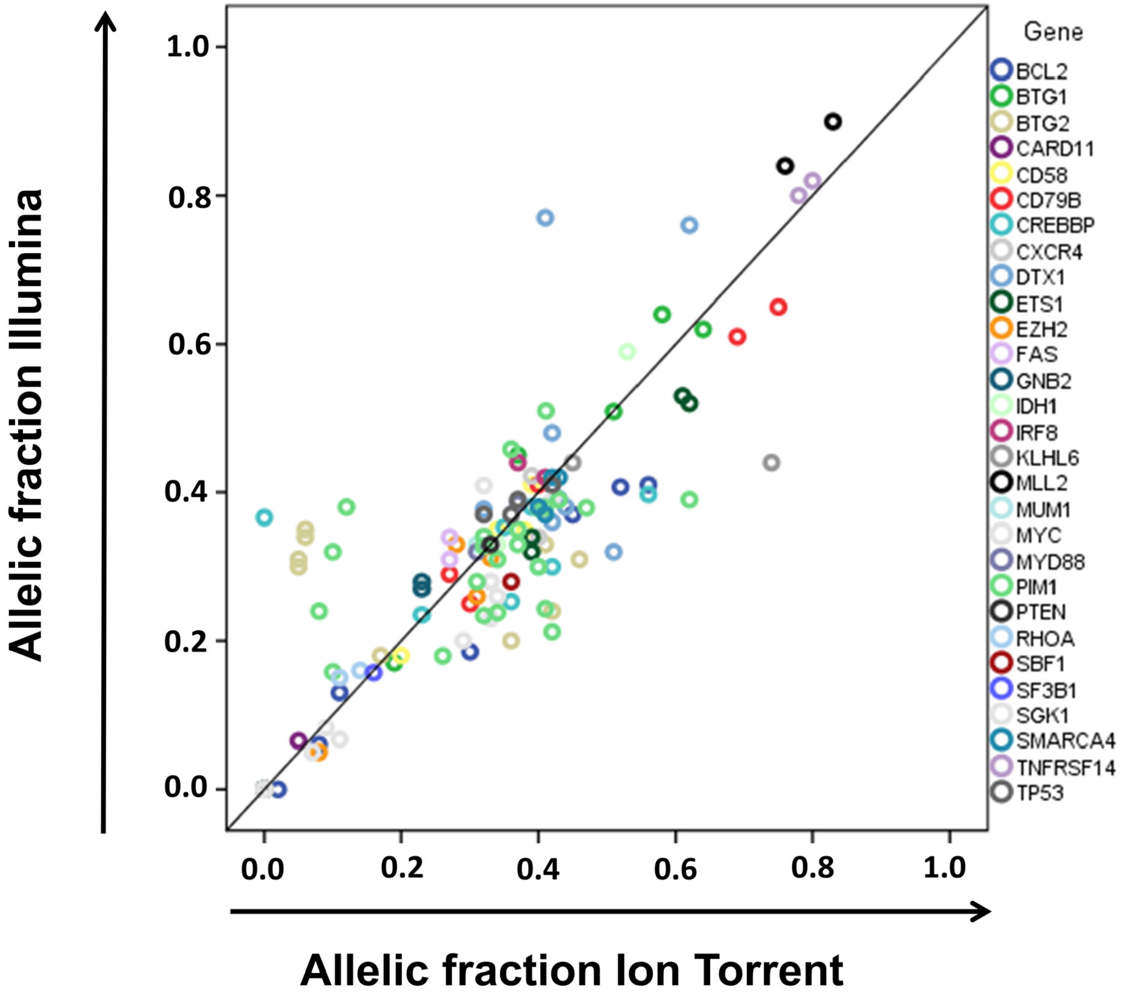

Supplementary Figure S2: Comparison of the allelic fractions between both sequencing approaches.

Supplementary Table S1: Clinical characteristics of all included patients

Pat-ID	Sex	First diagnosis	Age	ECOG	Initial stage	NCCN-Score	IPI-Score	Nodal disease	Extranodal disease	First Line regimen	PFS (months)	Time to second biopsy (months
1	female	2011	84	1	4	high	high	yes	GIT, BM	R-CHOP	4	5
2	male	2008	76	1	4	high	high-int	yes	no	R-CHOP	9	9
3	male	2008	76	1	1	high-int	low-int	yes	GIT	R-CHOP	2	2
4	male	1991	39	0	1	n.a.	n.a.	no	testis	CHOP	204	204
5	male	2004	43	0	2	low-int	low-int	yes	GIT	R-CEOP	20	20
6	male	2007	55	1	3	low-int	low-int	yes	no	R-CHOP	3	9
8	male	2011	70	0	4	low-int	low-int	no	paranasal sinus, skin	R-CHOP	6	6
11	male	2007	73	2	2	high-int	high-int	yes	GIT	R-CHOP	23	23
12	male	2006	72	0	4	low-int	low-int	no	skin	R-CHOP	23	41
13	female	2006	63	0	4	high-int	high-int	yes	no	R-CHOP	22	22
14	male	2005	60	0	2	low	low	yes	no	R-CHOP	10	10
17	male	2010	58	0	4	high-int	low-int	yes	BM	R-CHOP	8	11
18	female	2010	61	0	4	high	high-int	no	BM	R-CHOP	15	15
20	female	2006	60	0	3	low-int	low-int	yes	no	R-CHOP	20	20
21	male	2009	64	0	3	low-int	low-int	yes	no	R-CHOP	6	13
24	female	2006	68	1	3	high-int	high-int	yes	GIT	R-CHOP	21	21
27	male	2005	62	1	4	high-int	high	yes	testis	R-CHOP	8	8
30	male	2006	62	3	3	high-int	high-int	yes	no	R-CHOP	11	11
32	male	1997	55	1	2	low-int	low	yes	no	CHOP	147	147
36	male	2007	76	0	4	high	high	yes	lung	R-CHOP	4	4
37	male	2002	60	0	1	low	low	yes	no	CHOP	4	4
39	female	2005	22	0	4	low	low	no	skin	R-CHOP	80	80
40	male	2010	76	2	3	high	high	yes	no	R-CHOP	15	27
41	male	2007	56	2	3	high-int	high-int	yes	no	R-DHAP	2	17
43	male	2008	22	0	1	low	low	yes	no	CHOP	4	7
47	female	2012	61	0	4	high	high-int	yes	BM	R-CHOP	7	7
48	male	2009	69	0	1	low-int	low	yes	no	R-CHOP	45	45
49	male	2012	68	1	3	high-int	high-int	yes	bladder	R-CHOP	5	5

n.a.= not available; n.t.= not translocated; transl.= translocated; GCB=germinal center B-cell; BM= bone marrow, GIT= gastrointestinal tract

Supplementary Table S2: Immunohistochemistry and FISH analyses of all included patients

Pat-ID	Cell of origin	MYC	Bcl 2/6 rearrangement	CD10	bcl-2	bcl-6	MUM1	p53 protein expression	Ki67
1	GCB	n.t.	n.a.	positive	positive	positive	negativ	weak	60%
2	non-GCB	n.t.	n.a.	negativ	positive	negativ	positive	very weak	80%
3	GCB	n.t.	n.a.	positive	positive	positive	negativ	positive	75%
4	n.a.	n.a.	n.a.	n.a.	negativ	positive	n.a.	n.a.	80%
5	GCB	n.t.	n.a.	negativ	negativ	positive	weak	positive	65%
6	GCB	n.t.	n.a.	positive	positive	positive	negativ	weak	50%
8	non-GCB	n.t.	n.a.	negativ	positive	negativ	positive	weak	80%
11	non-GCB	n.t.	n.a.	negativ	positive	positive	positive	positive	55%
12	non-GCB	n.t.	n.a.	negativ	positive	positive	positive	weak	50%
13	GCB	n.t.	n.a.	positive	positive	positive	weak	positive	75%
14	non-GCB	n.t.	n.a.	negativ	positive	negativ	positive	negativ	70%
17	GCB	n.t.	n.a.	positive	positive	negativ	negativ	positive	85%
18	non-GCB	n.t.	n.a.	negativ	positive	negativ	positive	weak	75%
20	non-GCB	n.t.	n.a.	negativ	positive	negativ	weak	positive	25%
21	GCB	transl	Bcl2	positive	positive	positive	na	positive	60%
24	GCB	n.t.	n.a.	positive	positive	positive	negativ	positive	60%
27	non-GCB	n.t.	n.a.	negativ	positive	negativ	positive	weak	85%
30	n.a.	n.a.	n.a.	negativ	positiv	negativ	n.a.	n.a.	n.a.
32	non-GCB	n.t.	n.a.	negativ	positive	positive	positive	very weak	55%
36	non-GCB	transl	Bcl2	positive	positive	negativ	very weak	weak	65%
37	non-GCB	transl	no	negativ	positive	negativ	negativ	negativ	75%
39	GCB	n.t.	n.a.	negativ	positive	positive	negativ	negativ	85%
40	non-GCB	n.t.	n.a.	negativ	positive	negativ	positive	weak	80%
41	non-GCB	n.t.	n.a.	negativ	positive	positive	positive	positive	65%
43	non-GCB	n.t.	n.a.	negativ	very weak	positive	positive	positive	50%
47	GCB	n.t.	n.a.	positive	positive	positive	negativ	weak	55%
48	non-GCB	transl	Bcl2 and Bcl6	negativ	positive	positive	positive	weak	65%
49	non-GCB	n.t.	n.a.	negativ	positive	positive	negativ	positive	45%

n.a.= not available;

Supplementary Table S3: Frequency of mutated genes (non-synonymous mutations) in primary and relapse samples

GENE	Mutated primary samples	Mutated relapsed samples	Mutated primary cases in the literature
**B2M**	1 of 25 (4%)	2 of 27 (7%)	32 of 400 (8%)
**CARD11**	3 of 25 (12%)	3 of 27 (11%)	62 of 458 (14%)
**CD79B**	2 of 25 (8%)	3 of 27 (11%)	45 of 458 (10%)
**CREBBP**	5 of 25 (20%)	3 of 27 (11%)	72 of 458 (16%)
**EZH2**	4 of 25 (16%)	7 of 27 (26%)	62 of 454 (14%)
**MYD88**	3 of 25 (12%)	4 of 27 (15%)	53 of 458 (12%)
**PIM1**	9 of 25 (36%) *	9 of 27 (33%) *	67 of 400 (17%)
**TP53**	7 of 25 (28%)	9 of 27 (33%) *	73 of 458 (16%)
**CD58**	2 of 25 (8%)	3 of 27 (11%)	19 of 320 (6%)
**MLL2**	11 of 25 (44%) *	11 of 27 (41%)	89 of 366 (24%)
**GNA13**	3 of 25 (12%)	2 of 27 (7%)	53 of 347 (15%)
**BCL2**	9 of 25 (36%) *	11 of 27 (41%) *	74 of 400 (19%)
**SMARCA4**	4 of 25 (16%) *	4 of 27 (15%) *	none reported
**NOTCH1**	4 of 25 (16%) *	3 of 27 (11%)	6 of 160 (4%)
**RB1**	4 of 25 (16%) *	6 of 27 (22%) *	2 of 89 (2%)
**MYC**	5 of 25 (20%) *	6 of 27 (22%) *	12 of 233 (5%)
**FAT2**	3 of 25 (12%) *	5 of 27 (19%) *	4 of 176 (2%)
**ATM**	3 of 25 (12%) *	4 of 27 (15%) *	none reported
**MCL1**	2 of 25 (8%) *	3 of 27 (11%) *	none reported

* indicates a statistically significant difference ($p<0.05$) compared to the literature

Supplementary Table S4: Genes used for targeted sequencing

ARID1A	CDKN2B	IKBKE	PDGFRA
ARID1B	CHD2	IKZF1	PDGFRB
ARID2	CIITA	IKZF2	PIK3CA
ARID3A	CREBBP	IKZF3	PIK3CD
ASXL1	CTSS	IRF4	PIM1
ATM	CXCR4	IRF8	PTEN
B2M	DIRAS3	KAT2A	RB1
BCL2	DNMT3A	KAT2B	RET
BCL7A	DTX1	KAT5	RHOA
BIRC3	EP300	KDM6A	ROS1
BRAF	EPHA6	KLHL6	SBF1
BRWD3	EPHA7	MALT1	SF3B1
BTG1	ETS1	MCL1	SGK1
BTG2	ETV6	MDM2	SIN3A
CARD11	EZH2	MEF2B	SMARCA2
CCND1	FAS	MLL2	SMARCA4
CCND2	FAT2	MUM1	SMARCB1
CCND3	FBXO11	MYC	STAT6
CD40	FOXO1	MYD88	SWAP70
CD58	GNA13	NF1	TET2
CD79A	GNB1	NOTCH1	TNFAIP3
CD79B	GNB2	NOTCH2	TNFRSF14
CDK4	ID3	NPM1	TP53
CDK6	IDH1	P2RY8	TRAF2
CDKN2A	IDH2	PAX5	TRAF3
			TRAF6
			TYK2
			UBR5
			USP6

Supplementary Table S5: Primers used for ultra-deep sequencing

CREBBP_10_FOR	GTCTGTTATATTATCACTTATTAGCAGAG	CREBBP_10_REV	GTCTGTTTTACTTGGAGGTCTCACAGGTT
CREBBP_25_FOR	GAACCTGCACCTTGTCAGCAACAGCCTTT	CREBBP_25_REV	GAACCTGGACATCCACGCCGTCAATTTCC
CARD11_3_FOR	CTAACTACGCCTTGTGGGAGAATGTGGAG	CARD11_3_REV	CTAACTAGCAGCATAGGGGCATTAAGCAC
CARD11_8_FOR	CATCGGTGTGTGGTTCACAGTACCTGGAG	CARD11_8_REV	CATCGGTGGTGCCTGTACCTGGTCCCGCT
p53_5_FOR	TCGACTCCTCTGTCTCCTTCCTCTTCC	p53_5_REV	TCGACTCGCAATCAGTGAGGAATCAGAGG
p53_7_1_FOR	GATTCACCTGCTTGCCACAGGTCTCC	p53_7_1_REV	GATTCACAAGCCACAGGTTAAGAGGTCCC
p53_7_2_FOR	GCACGAACTGCTTGCCACAGGTCTCC	p53_7_2_REV	GCACGAAAAGCCACAGGTTAAGAGGTCCC
p53_7_3_FOR	TTACCGTCTGCTTGCCACAGGTCTCC	p53_7_3_REV	TTACCGTAAGCCACAGGTTAAGAGGTCCC
p53_7_4_FOR	TGATGCGCTGCTTGCCACAGGTCTCC	p53_7_4_REV	TGATGCGAAGCCACAGGTTAAGAGGTCCC
p53_7_5_FOR	AGACCGTCTGCTTGCCACAGGTCTCC	p53_7_5_REV	AGACCGTAAGCCACAGGTTAAGAGGTCCC
p53_7_6_FOR	CTGCAATCTGCTTGCCACAGGTCTCC	p53_7_6_REV	CTGCAATAAGCCACAGGTTAAGAGGTCCC
p53_8_1_FOR	TTCAATGCTCCAGAAAGGACAAGGGTGG	p53_8_1_REV	TTCAATGGTTGGGCAGTGCTAGGAAAGAG
p53_8_2_FOR	TGCGTAGCTCCAGAAAGGACAAGGGTGG	p53_8_2_REV	TGCGTAGGTTGGGCAGTGCTAGGAAAGAG
p53_8_3_FOR	AGAACACCTCCAGAAAGGACAAGGGTGG	p53_8_3_REV	AGAACACGTTGGGCAGTGCTAGGAAAGAG

Supplementary Table S6: Detected mutations of genes frequently reported in DLBCL

Gene	Effect	Protein Change	Cosmic annotated
CARD11	Missense Mutation	p.D357V	yes
CARD11	Missense Mutation	p.C49Y	yes
CARD11	Missense Mutation	p.T732I	no
CARD11	Missense Mutation	p.R223Q	no
CD79B	Missense Mutation	p.Y197C	yes
CD79B	Missense Mutation	p.Y197D	yes
CD79B	Missense Mutation	p.Y197S	yes
CD79B	Missense Mutation	p.S76R	no
CD79B	Splice Site Mutation	p.D185_splice	no
EZH2	Missense Mutation	p.Y646S	yes
EZH2	Missense Mutation	p.Y646N	yes
EZH2	Missense Mutation	p.Y646F	yes
EZH2	Missense Mutation	p.Y646H	yes
IRF8	Frame Shift Mutation	p.A393fs	no
IRF8	Nonsense Mutation	p.E420*	no
IRF8	Missense Mutation	p.Y23F	no
IRF8	Nonsense Mutation	p.Q17*	no
IRF8	Nonsense Mutation	p.C2R	no
IRF8	Missense Mutation	p.Y23H	no
MYD88	Nonstop Mutation	p.*205R	yes
MYD88	Missense Mutation	p.S219C	yes
TP53	Missense Mutation	p.C176F	yes
TP53	Missense Mutation	p.Y163D	yes
TP53	Missense Mutation	p.Y234H	yes
TP53	Missense Mutation	p.I195T	yes
TP53	Missense Mutation	p.N239S	yes
TP53	Missense Mutation	p.L257R	yes
TP53	Missense Mutation	p.R248Q	yes
TP53	Missense Mutation	p.R273H	yes
TP53	Missense Mutation	p.G245S	yes
TP53	Missense Mutation	p.R282W	yes
TP53	Nonstop Mutation	p.*347K	no
